# Leukotriene A4 hydrolase (*LTA4H* rs17525495) gene polymorphisms and paradoxical reactions in extrapulmonary tuberculosis

**DOI:** 10.1038/s41598-023-30923-2

**Published:** 2023-03-06

**Authors:** Selwyn Selva Kumar, Raja Solomon, Priyanka Gautam, Leeberk Raja Inbaraj, Ajith Sivadasan, Joy Sarojini Michael, Rajiv Karthik, George M. Varghese, Christhunesa S. Christudass, Prasanna Samuel, Abi Manesh

**Affiliations:** 1 Department of Infectious Diseases, Christian Medical College, Tamil Nadu, Vellore, 632004 India; 2 Division of Neurochemistry, Department of Neurological Sciences, Christian Medical College, Vellore, Tamil Nadu India; 3 Department of Clinical Research, ICMR-National Institute for Research in Tuberculosis, Chennai, Tamil Nadu India; 4 Division of Neurology, Department of Neurological Sciences, Christian Medical College, Vellore, Tamil Nadu India; 5 Department of Microbiology, Christian Medical College, Vellore, Tamil Nadu India; 6 Department of Biostatistics, Christian Medical College, Vellore, Tamil Nadu India

**Keywords:** Health care, Medical research

## Abstract

Paradoxical reactions (PRs) are poorly studied complex immunological phenomena, among patients with tuberculosis (TB). When PRs involves critical structures like the central nervous system (CNS), immunomodulatory therapy is often required. Predictors for PRs in TB to pre-empt appropriate treatment strategies in high-risk groups are lacking. TT genotype of Leukotriene A4 hydrolase (*LTA4H*) promoter region rs17525495 polymorphisms are associated with exaggerated immune responses in Tuberculous meningitis (TBM), the most severe form of extrapulmonary tuberculosis (EPTB). The association of these polymorphisms with PRs is not known. We evaluated this plausibility among 113 patients with EPTB, at high risk of PRs. Majority [81 (71.7%)] had disseminated tuberculosis with prominent CNS [54 (47.8%)] and lymph node involvement [47 (41.6%)]. Human immunodeficiency Virus (HIV) co-infection was seen among 23 (20.3%) patients. PRs were noted in 38.9% patients, at a median duration of 3 months (IQR 2–4). *LTA4H* rs17525495 single nucleotide polymorphism (SNP) analysis showed 52 (46%) patients had CC, 43 (38.1%) had CT and 18 (15.9%) had TT genotypes. There was no statistically significant difference in occurrence [CC 38.5% vs CT 39.5% vs TT 38.7%] and time of onset [median (IQR)] of PRs across the genotypes [CC 3 (1–4.7), CT 3 (2–5), TT 2 (2–3)]. PRs was shown to be significantly linked with HIV co-infection (RR 0.6, 95% CI 0.29–1.28), culture positivity (RR 0.5, 95% CI 0.28–1.14), TB Lymphadenitis (RR 0.7, 95% CI 0.44–1.19) and CNS involvement RR 2.1, 95% CI 1.27–3.49) in the univariate analysis (p < 0.2). On multivariate analysis, CNS involvement alone was associated with PRs (aRR 3.8 (1.38–10.92); p < 0.01). PRs were associated with CNS involvement but not with *LTA4H* rs17525495 polymorphisms.

## Introduction

Paradoxical reaction refers to worsening of pre-existing tubercular lesions or appearance of new lesions after initial clinical improvement while on appropriate antitubercular therapy (ATT). It complicates up to a third of patients with EPTB and remains poorly studied^1^. The underlying mechanism appears to be immunologically mediated and variable in timing of occurrence, duration and severity. Most patients with PRs involving critical areas like the CNS require immunomodulatory therapy like steroids and upfront identification of these patients is important. The unpredictable nature of the PRs makes clinicians suspect drug resistance, therapy failure and alternative diagnosis. This makes PRs difficult to study and it remains an under researched area. Upfront identification of these patients can help clinicians to optimise therapy avoiding further diagnostic evaluation. Reported risk factors in the literature include younger age, female gender, shorter duration of symptoms, HIV co-infection, lower blood lymphocyte count, disseminated disease, acid fast bacilli positivity on smear and worsening of cerebrospinal fluid (CSF) parameters among patients with CNS tuberculosis^2,3^. However, most of these risk factors are non-specific. Recently, a SNP in the promoter region of the *LTA4H* rs17525495 (TT) was shown to predict hyperinflammatory cytokine phenotype and response to immunomodulation in TBM survival and severe immune reconstitution and inflammatory syndrome in HIV associated pulmonary TB^4–6^. There is biological plausibility for this association as *LTA4H* is a key enzyme in eicosanoid synthesis modulating the balance between its products—pro-inflammatory Leukotriene B4 (LTB4) and anti-inflammatory Lipoxin A4 (LXA4)^7^. We hypothesised that hyper inflammatory genotype of the *LTA4H* polymorphism (TT) may be associated with PRs. The incidence of PRs is not equally distributed among patients with EPTB. Patients with disseminated disease, CNS and nodal involvement are particularly high risk for PRs, providing enriched group to study this hypothesis. This may provide the first genomic test to predict PRs, identifying a group of patients who require pre-emptive immunomodulatory treatment.

## Results

Our study population included 113 adults, predominantly males (54.9%) from South (44.2%) and East India (55.8%) (Table 1). The mean age of our patients was 33.8 ± 11.7 years. One in five (20.4%) patients had HIV co-infection. Most of the study population (73.4%) had microbiologically confirmed TB. Rest of the patients had clinical features of TB supported by either histopathological or radiological evidence. Eleven patients had resistant TB (5 had multidrug resistant TB, 5 had isoniazid mono-resistance, 1 had Pyrazinamide mono-resistance). Disseminated TB (71.7%) was most common, followed by CNS TB (47.8%) and TB lymphadenitis (41.6%). In those with TBM (n = 54), 77.5% fulfilled Lancet consensus scoring criteria for definite or probable TBM. Eight (14.8%) had Medical Research Council (MRC) grade-3 disease of TBM.

**Table 1:** Multivariate logistic regression analysis for predictors of paradoxical reactions.

Variable	Total (N = 113)	Paradoxical reactions present (n = 44)	Paradoxical reactions absent (n = 69)	Univariate analysis		Multivariate analysis	
				RR (95% CI)	P value	aRR (95% CI)	P value
Age (Mean ± SD)	33.8 ± 11.7	32.3 ± 11.3	34.8 ± 11.8	–	0.226	–	–
Sex							
Male (%)	62 (54.9)	24 (54.5)	38 (55.1)	–	0.956	–	–
Female (%)	51 (45.1)	20 (45.5)	31 (44.9)				
HIV positive* (%)	23 (20.4)	6 (13.6)	17 (24.6)	0.6 (0.29–1.28)	0.157	0.6 (0.23–2.05)	0.513
Regional variations							
South India (%)	50 (44.2)	20 (45.5)	30 (43.5)	–	0.837	–	–
East India (%)	63 (55.8)	24 (54.5)	39 (56.5)				
Xpert positive (%)	81 (71.7)	33 (75)	48 (69.6)	–	0.532	–	–
Culture positive^+^ (%)	28 (24.8)	7 (15.9)	21 (30.4)	0.5 (0.28–1.14)	0.081	0.4 (0.16–1.23)	0.120
Resistance (%)	11 (9.7)	6 (13.6)	5 (7.2)	–	0.264	–	–
Disseminated TB (%)	81 (71.7)	33 (75)	48 (69.6)	–	0.532	–	–
TB lymphadenitis^#^ (%)	47 (41.6)	15 (34.1)	32 (46.4)	0.7 (0.44–1.19)	0.196	1.4 (0.52–4.26)	0.451
Abdominal involvement (%)	22 (19.5)	11 (25)	11 (15.9)	–	0.236	–	–
CNS involvement (%)	54 (47.8)	29 (65.9)	25 (36.2)	2.1 (1.27–3.49)	0.002	3.8 (1.38–10.92)	0.010
TBM brain alone (%)	33 (61.1)	16 (36.4)	17 (24.6)	–	0.495	–	–
TBM spinal cord (%)	6 (11.1)	3 (6.8)	3 (4.3)				
TBM brain + SC (%)	15 (27.8)	10 (22.7)	5 (7.2)				
Lancet criteria (n = 40)	40 (35.4)	24 (54.4)	16 (23.1)	–	–	–	–
Lancet-1 (Definite) (%)	9 (22.5)	4 (16.7)	5 (31.3)	–	0.350	–	–
Lancet-2 (Probable) (%)	22 (55)	13 (54.2)	9 (56.3)				
Lancet-3 (Possible) (%)	9 (22.5)	7 (29.2)	2 (12.5)				
MRC grades^+^ (n = 54)	54 (47.8)	29 (65.9)	25 (36.2)	–	–	–	–
MRC grade-1 (%)	20 (37)	9 (31)	11 (44)	–	0.599	–	–
MRC grade-2 (%)	26 (48.1)	15 (51.7)	11 (44)				
MRC grade-3 (%)	8 (14.8)	5 (17.2)	3 (12)				
CC genotype (%)	52 (46)	20 (45.5)	32 (46.4)	–	0.994	–	–
CT genotype (%)	43 (38.1)	17 (38.6)	26 (37.7)				
TT genotype (%)	18 (15.9)	7 (15.9)	11 (15.9)				
Sub optimal dosing of ATT (%)	25 (22.1)	11 (25)	14 (20.3)	–	0.556	–	–

^+^Two out of 28 culture positives were Xpert negative.

^#^TB lymphadenitis includes intra-abdominal lymphadenopathy.

Other sites involved were bone (11), spleen (6), pleura (4), eye (4), psoas abscess (3), epididymis (2), skin (1), pericardium (1), pyometra (1), renal abscess (1), prostatic abscess (1).

Patients are counted more than in the presentation of manifestations.

More than one third (38.9%) of our patients developed PRs at 3 months (IQR 2–4) after initiating ATT. PRs resulted in modification of therapy in 28 (63.6%) patients in the form of modification of steroid dose or administration of immunomodulators other than steroids. No patient had change in ATT in view of PRs. Overall, CC genotype (46%) was found more common than CT (38.1%) and TT (15.9%) genotypes. In the univariate analysis, HIV co-infection (RR 0.6, 95% CI 0.29–1.28), culture positivity (RR 0.5, 95% CI 0.28–1.14), TB lymphadenitis (RR 0.7, 95% CI 0.44–1.19) and CNS involvement (RR 2.1, 95% CI 1.27–3.49) were found to be significantly associated with PRs. However, when these predictors were taken into multivariate model, only CNS involvement had almost fourfold [aRR (95% CI), 3.8 (1.38–10.92), P = 0.010] higher risk of developing PRs than their counterparts.

Of the 23 patients with HIV, 19 had Xpert positive results, of which 5 (26.3%) showed PR while 14 (73.7%) did not show PR. Among the four patients with Xpert negative results, only 1 (25%) showed PR while 3 (75%) did not show PR. This finding was found statistically insignificant with p = 1.000. Eight percent of patients were non-adherent to the ATT treatment where 44.4% of them showed PR and 55.6% did not show PR (p = 0.734).

We also analysed the distribution of *LTA4H* rs17525495 polymorphism genotypes (CC, CT, TT) with the baseline characteristics and outcomes (Table 2). We found there was no significant association between baseline characters and genotypes including culture positivity (CC 23.1% vs CT 30.2% vs TT 16.7%) and severity of TBM. We noted no statistically significant association in the occurrence (CC 38.5% vs CT 39.5% vs TT 38.7%) or time of onset [median (IQR)] [CC 3 (1–4.7), CT 3 (2–5), TT 2 (2–3)] of PRs and the genotypes. Three patients out of 113 died, two had CNS TB with CC genotype and one with CT genotype.

**Table 2:** Characteristics of patients with extrapulmonary tuberculosis, by *LTA4H* rs17525495 genotype.

Variable	Total (N = 113)	CC genotype (n = 52)	CT genotype (n = 43)	TT genotype (n = 18)	P value
Age (Mean ± SD)	33.86 ± 11.7	34.06 ± 12.8	34.72 ± 11.1	31.22 ± 9.4	0.563
Sex					
Male (%)	62 (54.9)	33 (63.5)	21 (48.8)	8 (44.4)	0.226
Female (%)	51 (45.1)	19 (36.5)	22 (51.2)	10 (55.6)	
HIV status, HIV positive (%)	23 (20.4)	11 (21.2)	8 (18.6)	4 (22.2)	0.932
Regional variations					
South India (%)	50 (44.2)	26 (50)	18 (41.9)	6 (33.3)	0.435
East India (%)	63 (55.8)	26 (50)	25 (58.1)	12 (66.7)	
Xpert positive (%)	81 (71.1)	34 (65.4)	36 (83.7)	11 (61.1)	0.079
Culture positive (%)*	28 (24.8)	12 (23.1)	13 (30.2)	3 (16.7)	0.496
Resistance (%)	11 (9.7)	4 (7.7)	6 (14)	1 (5.6)	0.478
Disseminated TB (%)	81 (71.7)	33 (63.5)	34 (79.1)	14 (77.8)	0.200
TB Lymphadenitis (%)^#^	47 (41.6)	19 (36.5)	20 (46.5)	8 (44.4)	0.596
Abdominal involvement (%)	22 (19.5)	9 (17.5)	10 (23.3)	3 (16.7)	0.727
CNS involvement (%), n = 54	54 (47.8)	19 (36.5)	24 (55.8)	11 (61.1)	0.081
TBM brain alone (%)	33 (61.1)	11 (57.9)	14 (58.3)	8 (72.7)	0.928
TBM spinal Cord (%)	6 (11.1)	2 (10.5)	3 (12.5)	1 (9.1)	
TBM brain + SC (%)	15 (27.8)	6 (31.6)	7 (29.2)	2 (18.2)	
Lancet criteria (n = 40)					
Lancet-1 (Definite) (%)	9 (22.5)	2 (12.5)	7 (38.9)	0	0.108
Lancet-2 (Probable) (%)	22 (55.5)	10 (62.5)	9 (50)	3 (50)	
Lancet-3 (Possible) (%)	9 (22.5)	4 (25)	2 (11.1)	3 (50)	
MRC grades (n = 54)					
MRC grade-1 (%)	20 (37)	7 (36.8)	7 (29.2)	6 (54.5)	0.218
MRC grade-2 (%)	26 (48.1)	7 (36.8)	14 (58.3)	5 (45.5)	
MRC grade-3 (%)	8 (14.8)	5 (26.3)	3 (12.5)	0	
Paradoxical reactions, PRs (%)	44 (38.9)	29 (38.5)	17 (39.5)	7 (38.7)	0.994
Paradoxical reactions in Brain alone (%)	24 (54.5)	10 (50)	10 (58.8)	4 (57.1)	0.856
Paradoxical reactions in Spinal Cord alone (%)	13 (29.5)	7 (35)	5 (29.4)	1 (14.3)	0.586
Paradoxical reactions in Lymph node (%)	16 (36.4)	7 (35)	6 (35.3)	3 (42.9)	0.927
Paradoxical reactions as Tuberculoma (%)	23 (52.3)	10 (50)	9 (52.9)	4 (57.1)	0.946
Paradoxical reactions as Infarct (%)	15 (34.1)	5 (25)	8 (47.1)	2 (28.6)	0.349
Paradoxical reactions as Hydrocephalus (%)	9 (20.5)	3 (15)	5 (29.4)	1 (14.3)	0.505
Paradoxical reactions as Arachnoiditis (%)	6 (13.6)	4 (20)	1 (5.9)	1 (14.3)	0.459
Median duration of Paradoxical reactions from ATT initiation (IQR), in months	3 (2–4)	3 (1–4.7)	3 (2–5)	2 (2–3)	0.745

*Two out of 28 culture positives were Xpert negative.

^#^TB Lymphadenitis includes intra-abdominal lymphadenopathy.

## Discussion

PR often complicates patients with EPTB on ATT. In our study focussing on patients at high risk of PR, its prevalence was 38.9% and *LTA4H* rs17525495 polymorphisms were not related to their occurrence. TT genotype of *LTA4H* rs17525495 polymorphism is linked to hyperinflammation and the benefit of immunomodulatory therapy in CNS TB. This is the first study globally to evaluate whether these genotypes are associated with PRs. PRs are compartmentalised immunological phenomena, often without systemic symptoms. Hypothetically, these may be driven by complex interplay between the pathogen and host response in immunologically privileged areas like CNS, while *LTA4H* rs17525495 genotypes may have a broad role modulating immune responses at the organism level.

Close to two thirds (63.6%) of patients with PRs required immunomodulatory therapy. This is reflective of the large proportion of patients with CNS involvement in the study. While PRs can be self -limiting in milder forms of EPTB, it can be severe requiring therapy modification in others. CNS involvement alone was found to be high risk for the development of PRs. This observation may have implications—biomarkers in the blood may not reflect these immunological changes and evaluation of representative samples are needed, as reported by others^8^. Despite increased occurrence of PRs among people living with HIV (84.6%), it did not reach statistical significance, probably due to type II error^9^.

While the global distribution of rs17525495 SNP genotypes is similar across the Vietnamese, Indonesian, Zambian and our study cohorts, important differences exist in the in vivo and in vitro observations^5,10,11^. Zebrafish larvae modelling studies show the *LTA4H* gene regulates inflammatory milieu by controlling the balance between pro- and anti-inflammatory eicosanoids and Tumor necrosis factor-alpha (TNF-α) production. The homozygous genotypes (CC and TT) are associated with accelerated bacterial growth in the larval stages while the heterozygotes (CT) are protected from severe *Mycobacterium marinum* infection^7^. However, the picture is more complex in humans. In large TBM cohorts, heterozygosity (CT) corresponds to balanced inflammatory response whereas homozygosity (CC/TT) represents polar spectrums of disease. CC correlates to hypo-inflammation with low TNF alpha levels and increased bacterial burden while TT with high TNF alpha levels, controlled mycobacterial burden and hyperinflammatory host immune response^5^. TT genotype predicted high pro-inflammatory cytokine milieu in the CSF and the response to corticosteroids among HIV uninfected patients in Vietnam but not in Indonesia and Zambia^5,10,11^. Among HIV-pulmonary TB coinfected patients, TT genotype was associated with severe IRIS phenomena^6^. Similar association was not reported in the Vietnamese cohort with CNS TB^5^.

Our study cohort has important differences from those published in the literature. Few studies have evaluated PR in many types of EPTB^3^. We had high rates of disseminated disease, CNS involvement and patients requiring immunomodulation as we preferentially recruited patients at high risk of PR. The rates of PR in our study were similar to TBM cohorts but lesser than cohorts with several types of EPTB^2,9^. The median duration of PR in our study was similar to TBM cohorts but longer than other EPTB data^3,9^.

What could explain such apparent diverse observations? Firstly, PRs may be caused by local immune responses in immune sanctuary sites like CNS and are not controlled by host genotypes. This is also supported by paucity of systemic response like fever or elevated inflammatory markers in non-HIV infected patients with PRs. Though majority of our patients had disseminated tuberculosis, the PRs are often localised to a single organ^2,3,9^. Alternatively, the effect of *LTA4H* polymorphisms may be modulated by other gene systems and epigenetic factors in various populations. Studies reporting on *LTA4H* polymorphisms could consider measuring the gene products while evaluating their association with clinical outcomes. Thirdly, significant variability across regions may be due to population genetic factors like “Linkage Disequilibrium”^12^. Linkage disequilibrium is the non-random association of alleles at different loci in diverse populations. Within ethnic groups, certain genetic loci or alleles of interest are conserved and remain unchanged in a particular position. This may lead to erroneous implication of a tested SNP allele as the cause of a phenotype when, a second unknown SNP with alleles strongly correlated with the tested one is actually responsible. This may explain the difference in association of *LTA4H* SNPs with TB survival in the Indonesian and Vietnamese population^13,14^. Our study shows TT genotype had a trend towards lesser culture positivity (16.7%) compared to hypo-inflammatory CC type (23.1%), suggesting the potential role of hyper-inflammatory TT genotype in restricting mycobacterial replication, probably due to excessive inflammation (Table 2). Similar findings were also observed in the Zambian study^10^.

Our study has several limitations. While the sample size was small, a significant number of patients developed PRs. It is a single centre study and the distribution of the genotypes of our cohort represents only a restricted topography. Since our study focussed on CNS and lymph nodal TB, the findings may not be applicable for all EPTB. In summary, PRs were associated with CNS involvement but not *LTA4H* rs17525495 polymorphisms in our study.

## Methods and material

We conducted a retrospective cohort study among adult patients with EPTB, at high risk of PR, in the Departments of Infectious Diseases and Neurological Sciences at Christian Medical College and Hospital, Vellore, India from 1st June, 2020 to 31st December, 2021. We recruited patients with disseminated, CNS and nodal involvement preferentially in view of higher incidence and clinical impact in these groups. We retrieved patient's clinical characteristics, laboratory findings, Mycobacterium tuberculosis genotypic and phenotypic resistance, treatment regimens, *LTA4H* genotypic status, imaging data and clinical or radiological evidence of PRs from the hospital electronic database. Tuberculosis in the study was defined as follows: patients with clinical features of TB with one or more of the following—(1) Microbiological evidence (mycobacterial culture positivity or Cepheid GeneXpert positive for *Mycobacterium tuberculosis*), (2) Histopathology showing necrotizing granulomatous inflammation, (3) Radiological evidence supporting TB (where tissue diagnosis is not feasible e.g. CNS TB) were included in the study. We defined disseminated tuberculosis as having two or more non-contiguous sites^15^. Patients who did not develop PRs were considered as controls. All the patients included in the study completed 3 months of follow-up, correlating with the period of PRs occurrence. During each visit, clinical improvement, PRs, weight of the patient and compliance on ATT were documented. Dose of ATT was optimised as per the weight of the patient at each visit. Non-adherence was defined as patients missing ≥ 10% of total prescribed dose^16^. ‘Modification of therapy’ in the study refers to one of the three after the onset of PRs—(1) Increase in the dose of steroids (2) Addition of steroids, or (3) Administration of immunomodulators other than steroids in addition to weight based anti-tuberculous therapy. The International Network for the Study of HIV-associated Immune reconstitution inflammatory syndrome [IRIS] (INSHI) consensus case definition was used to define PRs^17^. It requires a diagnosis of TB with an initial positive response to treatment, characteristic clinical features (such as new or enlarging lymphadenopathy, constitutional, respiratory or abdominal symptoms, or new or worsening radiological features), and exclusion of alternative explanations for clinical deterioration. Patients with TBM were classified as definite, probable and possible as per the Lancet Consensus scoring system for TBM diagnosis^18^. The management of PRs were decided by the respective treating physicians.

Genetic analysis for *LTA4H* rs17525495 polymorphisms were done as a part of routine care in our patients in line with the emerging association with outcomes in severe forms of tuberculosis^9^. Genomic DNA from the samples was isolated using Qiagen Mini DNA blood kit. The genotyping of SNP rs17525495 in these samples were done by a PCR–RFLP method developed by our Neurochemistry Laboratory and was validated previously using a commercially available TaqMan assay kit and Sanger sequencing. PCR, which yielded a product of 175 bp, was performed for 30 cycles using the primers 5′-TTAAGAAACTTCCTTTCCCGGC-3′ and 5′-CCAACGAACAGGTATCCACTAT-3′ with 58 °C as annealing temperature. The PCR products were digested with the restriction enzyme *HpyAV* (2.5 units/sample) for 6 h at 37 °C and were resolved in 2% agarose gel after digestion. As this enzyme cuts the sample with T allele into two fragments, we identified the CC genotype by the presence of an uncut 175 bp fragment, CT genotype by 175, 103 and 72 bp fragments and TT genotype by 103 and 72 bp fragments.

We also evaluated the association of PRs with multiple clinical parameters like organ involvement, presence of disseminated disease, mycobacterial culture positivity, HIV co-infection, drug resistant tuberculosis and suboptimal TB treatment.

The descriptive statistics of socio-demographic and other clinical parameters of the study population were analysed using frequency and percentages for categorical variables; mean and standard deviation (SD), median and inter quartile range (IQR) for continuous variables. Associations between PRs and clinical predictors were assessed using chi-square tests or Fisher’s exact tests as appropriate. An independent sample t-test was performed to find the mean difference between two groups with continuous variables. Factors with a p < 0.20 were considered for inclusion in the multivariate analysis. Multivariate Logistic Regression analysis was performed to assess the independent effects of factors associated with PRs. To account for the multiplicity of tests, a Bonferroni correction was applied taking a two-sided P-value of 0.01 as significance level. All analyses were performed using the software programs SPSS (version 23).

### Ethics declaration and consent to participate

Ethical approval was obtained from the Institutional Review Board (IRB Min No. 14456) of Christian Medical College and hospital, Vellore, Tamil Nadu, India. The IRB waived participant consent as this is a retrospective study. Additionally, all methods were performed in accordance with the relevant guidelines and regulations.

## Data Availability

Data are available upon reasonable request to the corresponding author.

## Abbreviations

*LTA4H*: Leukotriene A4 hydrolase
PRs: Paradoxical reactions
TBM: Tuberculous meningitis
EPTB: Extrapulmonary tuberculosis
TB: Tuberculosis
CNS: Central nervous system
HIV: Human immunodeficiency virus
ATT: Anti tuberculosis therapy
IQR: Interquartile range
CSF: Cerebrospinal fluid
SNP: Single nucleotide polymorphism
MRC grading: Medical Council of Research grading system for tuberculous meningitis
TNF-α: Tumor necrosis factor-alpha
IRIS: Immune reconstitution inflammatory syndrome
INSHI: International Network for the Study of HIV-associated Immune IRIS
SD: Standard deviation
SC: Spinal cord
